# Evaluating the Learning Curve and Patient Outcomes in Endoscopically Assisted Craniosynostosis Surgery: A 20-Year Analysis

**DOI:** 10.1097/SCS.0000000000010755

**Published:** 2024-10-11

**Authors:** Najiba Chargi, Mark Kregel, Tong Xi, Titiaan Dormaar, Wilfred Borstlap, Erik van Lindert, Hans Delye, Marloes Nienhuijs

**Affiliations:** * Department of Oral and Maxillofacial Surgery, Radboud University Medical Center; † Department of Neurosurgery, Radboud University Medical Center, Nijmegen, The Netherlands

**Keywords:** Craniosynostosis, endoscopy, helmet therapy, learning curve, minimal invasive

## Abstract

**Objectives:**

To analyze the learning curve associated with endoscopic-assisted craniosynostosis surgery (EACS) at a single institution over a period of 2 decades.

**Material and methods:**

Patients who underwent EACS between 2004 and 2023 were included in this retrospective study. The impact of surgical experience was assessed by analyzing the duration of surgery and anesthesia, blood loss, need for blood transfusion, postoperative complications, and length of hospital stay, in relation to the number of surgeries performed.

**Results:**

On the basis of 310 patients, the overall complication rate was low, with only 23 patients (7.4%) experiencing postoperative complications and 33 patients (10.6%) requiring a blood transfusion. The median length of hospital stay was 3 days (range 1–7 days). The results showed a statistically significant learning curve associated with EACS, with each additional surgery reducing the odds of postoperative complications by 0.7% (*P*<0.001) and the odds of blood transfusion by 0.8% (*P*<0.001). In addition, there were significant reductions in the duration of anesthesia, duration of surgery, and length of hospital stay over time (*P*<0.001).

**Conclusion:**

EACS is a safe and effective technique for treating craniosynostosis with low complication rates and a significant learning curve over time. Surgeons can expect to achieve better outcomes with greater surgical experience.

Craniosynostosis is a developmental disorder of the craniofacial region defined by the premature fusion of one or more cranial sutures. This premature fusion leads to characteristic head shapes and potential neurological complications resulting from the abnormal development of the brain.^1–4^ Craniosynostosis can manifest in various forms affecting one or multiple sutures and can occur in isolation or as part of a syndrome resulting from genetic mutations.^5^

Historically, surgical interventions for craniosynostosis have evolved significantly. The first surgical correction of craniosynostosis was described by Lane in 1890.^6^ In early treatments of craniosynostosis, strip craniectomies were performed, which involved the removal of the affected suture. However, postoperative refusion of the removed suture and unacceptable cosmetic outcomes were frequently observed, reducing the effect of surgery.^1^ Subsequent advancements in surgical and anesthetic techniques led to more invasive open surgical procedures involving removal, sectioning, and remodeling of the affected skull to correct the deformity.^7^ Although satisfactory results could be achieved with these operations, they were lengthy procedures with considerable morbidity, including high blood loss and prolonged postoperative stays.^7–9^

In recent decades, less invasive techniques have been explored to reduce the morbidity and invasiveness of conventional open surgery. The shift toward these minimally invasive techniques was catalyzed by the pioneering work of Jiminez and colleagues, who first reported successful outcomes of craniosynostosis treated with an endoscopic approach followed by postoperative orthotic helmet therapy.^10,11^ Using the endoscope, affected sutures can be surgically removed through limited skin incisions, minimizing tissue disruption and destruction. After surgery, orthotic helmet therapy plays a crucial role by directing the accelerated postoperative growth of the brain and skull. Unsatisfactory outcomes encountered after traditional treatment approaches are seldom observed after the contemporary treatment method.^1,10–12^

The Radboudumc Center of Expertise for Craniofacial Anomalies adopted this minimally invasive approach in 2004 and has become an extensively experienced provider of this treatment in the Netherlands.^13^ However, mastering this procedure requires years of supervised training.^14^ It is hypothesized that as surgical teams gain more experience, the efficacy and efficiency of their surgical outcomes improve.

Despite these advancements, there is little scientific evidence investigating the impact of surgical experience on endoscopic-assisted craniosynostosis surgery (EACS) outcomes. Therefore, this large sample size study aims to evaluate the effects of surgical experience on the outcomes of EACS and to identify a potential learning curve.

## MATERIAL AND METHODS

### Study Population and Variables

Patients diagnosed with craniosynostosis, who were surgically treated with EACS at the Radboud University Medical Center Nijmegen, between October 2004 and April 2023, were retrospectively included in this study. A subset of these patients has been included in our previous article in which we extensively described our EACS technique.^15^

Craniosynostosis was diagnosed clinically and confirmed radiologically using the institution ultra-low dose computed tomography (CT) scanning protocol with a conventional multislice spiral CT scan (Siemens Somatom 16).

The following patients’ characteristics and demographic variables were collected: sex, date of birth, age at the time of operation, length and weight of the patient, type of craniosynostosis, American Society of Anesthesiologists (ASA) classification, and presence of a syndrome.

The following outcome variables were collected: the duration of anesthesia (min), duration of surgery (min), intraoperative blood loss (ml), postoperative need for blood transfusion (yes/no), postoperative complications (yes/no), and the necessity of a second operation (yes/no).

### Surgical Procedure and Helmet Therapy

Initially, 2% lidocaine was used to infiltrate the skin, after which one or more small incisions were made depending on the affected suture(s) to access the skull bone. Monopolar cautery was then utilized to dissect the galea and periosteum. Once the cranium was exposed, a high-speed drill was used under copious irrigation to initiate the craniotomy, which was then continued using rongeurs and Kerrisons. Floseal (Baxter) and Ostene bone wax (Baxter) were used to control bleeding from the epidural space and bone edges. Subsequently, a 0-degree Storz lens scope was used to dissect the dura from the synostosis suture and the overlying bone. A separate aspirator was used for blood aspiration. The endoscope was used to continue the craniectomy along the entire length of the synostosis suture(s). After removal of the affected suture, the wound was closed in separate layers using resorbable sutures, and a small compressive head bandage was applied for 24 hours. A detailed description of the surgical procedure for different types of craniosynostosis is provided by Delye et al^16^ Postoperatively, the patients did not require admission to the ICU and were monitored in the pediatric medium care unit. After EACS, all patients underwent helmet therapy. The success of the surgery relies on the ability of the helmet to guide the 3-dimensional growth of the skull. By acting as a barrier in most areas, the helmet can focus cranial growth in areas where it is desired by using the brain as a natural, internal distractor. One week postoperatively, a plaster imprint of the patient’s skull was taken for fabrication of the custom-made helmet. The design of the helmet followed a strict pattern: all areas of the skull were contacted except for the areas where growth was desired. The orthosis had to have a perfect fit to prevent problems such as pressure ulcerations, slippage of the helmet, or skin problems. The helmet therapy started 2 weeks after surgery, with parents advised that the helmet should be worn 23 hours a day. Frequent follow-ups, especially at the beginning of the helmet therapy, were important to ensure perfect fit and patient-specific adjustments in response to the 3-dimensional growth of the skull. We advised helmet therapy to be continued until normocephaly was achieved. The mean duration of helmet therapy was 10 months and patients needed between 1 and 3 helmets throughout the complete therapy.^16^ A detailed description of the precise helmet design for the different types of craniosynostosis is given by Delye et al.^16^

### Statistical Analysis

In this study, IBM SPSS Statistics 27 was used for statistical analysis. Basic demographics were analyzed to describe the study population. Surgical procedures were chronologically numbered, and a scatter plot was generated to graphically analyze subcategories of craniosynostosis. The *x*-axis represented the chronological order of the surgeries, whereas the *y*-axis represented the outcome variables. These variables, which gave insight into the learning curve, included the duration of anesthesia (min), duration of surgery (min), intraoperative blood loss (ml), postoperative need for blood transfusion (yes/no), postoperative complications (yes/no), and necessity of a second operation (yes/no). The chronological order of surgery and the separate outcome variables were then analyzed using linear regression analysis for continuous variables and binary regression analysis for dichotomous variables, to investigate the effect of learning and determine its significance. The threshold for statistical significance was set at *P*<0.05.

## RESULTS

### Patients’ Characteristics

A total of 310 patients were included in the study. As shown in supplemental Table 1, Supplemental Digital Content 1, http://links.lww.com/SCS/G896, most patients were male (71.6%), had ASA I status (75.8%), did not have a syndrome (94.2%) and the most common type of craniosynostosis was scaphocephaly (51.6%). The median age at the time of surgery was 3.8 months, with an interquartile range (IQR) of 3.2 to 4.6 months.

The mean blood loss was 35.7 ml (standard deviation (SD): 31.6 ml), and the mean length of hospital stay was 1.3 days (SD 0.8 days). The median duration of anesthesia was 125.5 minutes (IQR 109–142 min), and the median duration of surgery was 53 minutes (IQR: 42–65 min).

Of all patients, 33 patients (10.6%) required a blood transfusion after their surgery, whereas 23 (7.4%) experienced postoperative complications. Furthermore, 10 patients (3.2%), including 3 patients with a syndrome, required a second operation. The complications that occurred were: dural tear in 10 patients (3.2%), local wound infection in 4 patients (1.3%), rupture of the sinus sagittalis superior in 4 patients (1.3%), liquour leak with wound dehiscence in 2 patients (0.6%), thrombocytosis (not further specified) in 1 patient (0.3%), and helmet related eczema in 1 patient (0.3%). The second operations performed were: 8 fronto-orbital advancements (2.6%), 1 biparietal open reconstruction (0.3%), and in 1 patient (0.3%) with Apert syndrome the re-operation (fronto-orbital advancement) was performed in another hospital.

### Learning Curve

#### Influence of Surgical Experience on Outcome Measures: Regression Analysis

**FIGURE 1:**
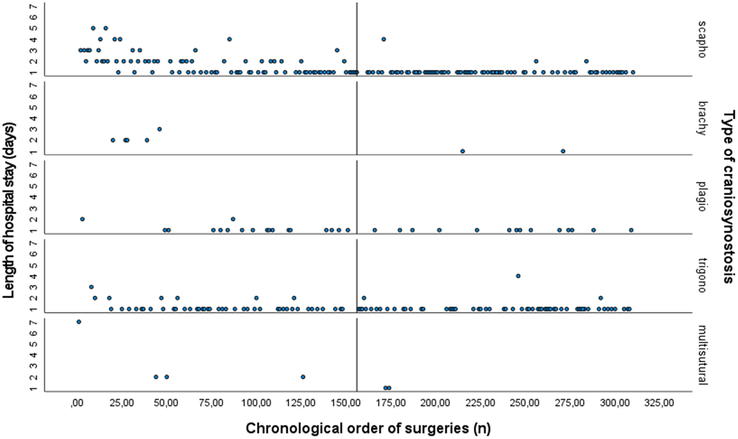
For each type of craniosynostosis: Scatter plot of the relationship between surgical experience as expressed as the chronological order of surgeries (surgery 0 till surgery 310) on the *x*-axis and the length of hospital stay (days) on the *y*-axis. The median surgery number (156^th^ surgery) is shown.

*Length of hospital stay*. Linear regression analysis showed that surgical experience, in terms of the number of previous surgeries performed, was a significant predictor of the length of hospital stay (Beta= −0.455, *P*<0.0001), as shown in supplemental Table 2, Supplemental Digital Content 1, http://links.lww.com/SCS/G896. This implies that for every additional surgery performed, the length of hospital stay decreased by 0.004 days, on average. The standardized coefficient (Beta) of −0.455 indicates that the inverse relationship between the number of surgeries and the length of stay was moderate to strong. Figure 1 illustrates the correlation between surgical experience and the reduction in hospital stay duration for each type of EACS.

**FIGURE 2:**
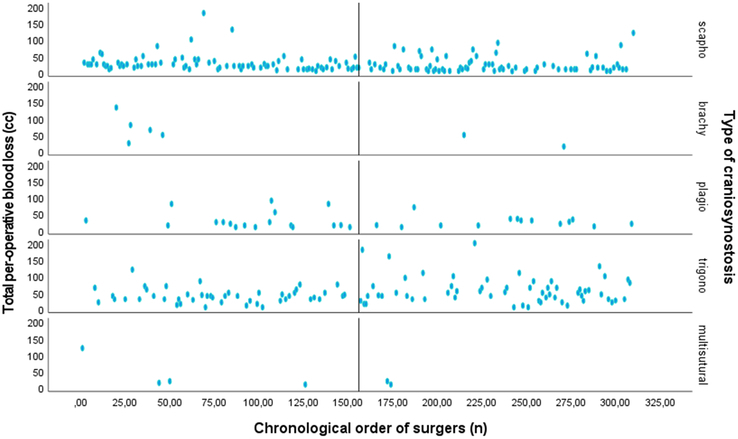
For each type of craniosynostosis: Scatter plot of the relationship between surgical experience as expressed as the chronological order of surgeries (surgery 0 till surgery 310) on the *x*-axis and the total per-operative blood loss (ml) on the *y*-axis. The median surgery number (156^th^ surgery) is shown.

*Total blood loss.* As shown in supplemental Table 2, Supplemental Digital Content 1, http://links.lww.com/SCS/G896, the surgical experience was not a statistically significant predictor of total blood loss (Beta=−0.007, *P*=0.908). A scatter plot (Fig. 2) depicts the correlation between surgical experience and total blood loss for each type of EACS.

**FIGURE 3:**
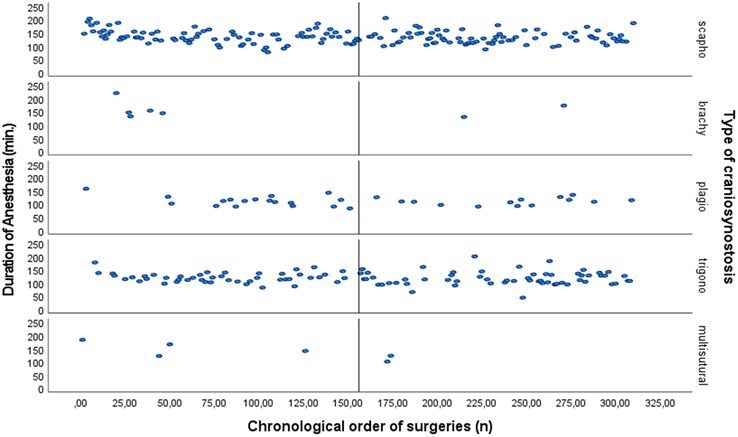
For each type of craniosynostosis: Scatter plot of the relationship between surgical experience as expressed as the chronological order of surgeries (surgery 0 till surgery 310) on the *x*-axis and the total duration of anesthesia (min) on the *y*-axis. The median surgery number (156^th^ surgery) is shown.

*Duration of anesthesia.* The results demonstrated a significant inverse correlation between surgical experience and the anesthesia duration (Beta=−0.188, *P*=0.001). As the number of performed surgeries increased, the anesthesia duration decreased. A scatter plot (Fig. 3), depicts the correlation between surgical experience and reduced duration of anesthesia for each type of EACS.

**FIGURE 4:**
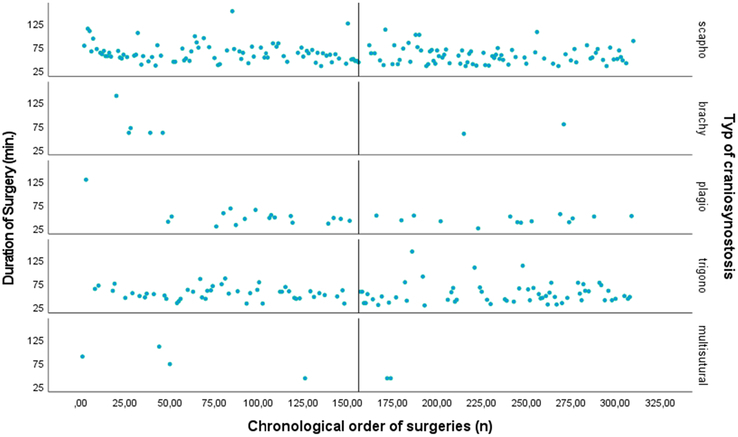
For each type of craniosynostosis: Scatter plot of the relationship between surgical experience as expressed as the chronological order of surgeries (surgery 0 till surgery 310) on the *x*-axis and the total duration of surgery (min) on the *y*-axis. The median surgery number (156^th^ surgery) is shown.

*Surgery duration.* The results also revealed an inverse correlation between surgical experience and the surgery duration (Beta=−0.199, *P*=0.0001). This indicates that with an increase in the number of surgeries performed, the duration of surgery tends to decrease proportionately. Figure 4, a scatter plot, illustrates this correlation, demonstrating a trend of reduced surgery duration with increased surgical experience for each type of EACS.

**FIGURE 5:**
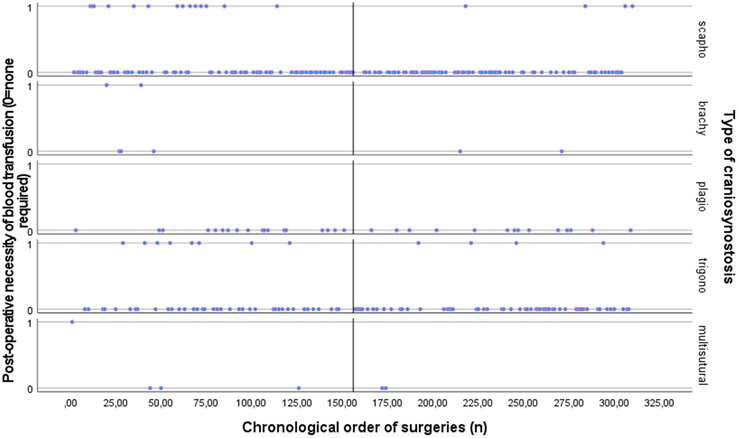
For each type of craniosynostosis: Scatter plot of the relationship between surgical experience as expressed as the chronological order of surgeries (surgery 0 till surgery 310) on the *x*-axis and the need of postoperative blood transfusion (0=none required, 1=required) on the *y*-axis. The median surgery number (156^th^ surgery) is shown.

*Need of blood transfusion.* As shown in supplemental table 3, Supplemental Digital Content 1, http://links.lww.com/SCS/G896 illustrated that surgical experience was a significant predictor of the need for blood transfusion (*P*<0.001). As the number of surgeries performed increased, the odds of needing a transfusion decreased (Exp (B) =0.992, 95% CI: 0.988–0.977), indicating that the odds of needing a blood transfusion decreased by 0.8% for each additional surgery performed. The correlation between surgical experience and the decreasing need for postoperative blood transfusions for each type of EACS is depicted in Figure 5.

**FIGURE 6:**
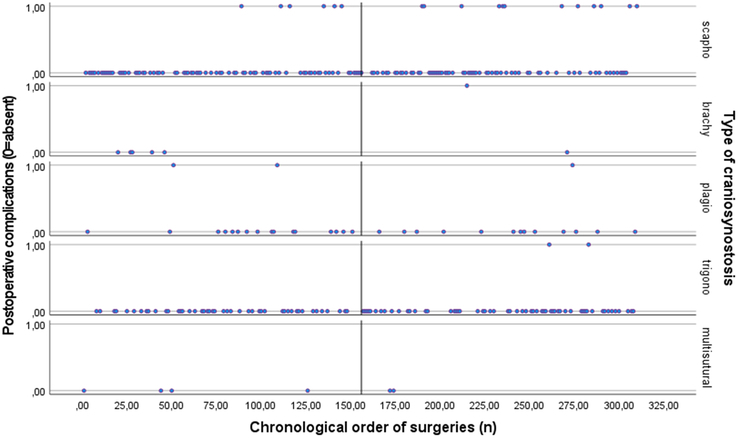
For each type of craniosynostosis: Scatter plot of the relationship between surgical experience as expressed as the chronological order of surgeries (surgery 0 till surgery 310) on the *x*-axis and occurrence of postoperative complications (0=absent, 1=present) on the *y*-axis. The median surgery number (156^th^ surgery) is shown.

*Postoperative complications.* As shown in supplemental table 3, Supplemental Digital Content 1, http://links.lww.com/SCS/G896 surgical experience significantly reduced the incidence of postoperative complications (B=−0.008, *P*=0.005). This suggests that with an increase in the number of surgeries performed, the log-odds of encountering postoperative complications decreased. The odds ratio (exp B) associated with a single-unit increase in surgery number was 0.993, indicating a 0.7% decrease in the odds of experiencing postoperative complications with each additional surgery performed. Figure 6, depicts the inverse correlation between surgical experience and the incidence of postoperative complications for each type of EACS.

## DISCUSSION

The results of this study show that EACS is a safe procedure with a low complication rate of 7.4% over a 20-year time period. The study also demonstrated a statistically significant learning curve associated with EACS, where an increase in the number of surgeries performed resulted in a decrease in the incidence of postoperative complications, need for blood transfusions, length of postoperative hospital stay, duration of anesthesia, and surgery time. Specifically, for every additional EACS performed, there is a 0.7% decrease in the odds of postoperative complications and a 0.8% decrease in the odds of blood transfusions.

These findings are consistent with previous studies that have demonstrated the safety and efficacy of EACS in treating craniosynostosis. Goyal et al^8^ noted a significant decrease in both operative and postoperative morbidity with EACS, as compared with traditional open surgery.

Comparatively, literature presents similar studies sharing their EACS surgical experiences. A study by Jimenez and colleagues presented their 16-year experience in EACS for sagittal synostosis. They reported that EACS was associated with low rates of blood loss, transfusions, and length of hospital stays.^15^ Another study by Dalle Ore and colleagues also presented their 16-year EACS experience on all types of craniosynostosis. They had a slightly higher complication rate of 7.7% and a higher blood transfusion rate of 50.2% compared with our study (transfusion rate of 10.6%).^17^

Our study stands out as the first to investigate the learning curve of EACS, in contrast to previously mentioned studies that primarily focused on surgical experience. These new insights have considerable implications for surgical training, emphasizing the need for high-volume centers and specialized training programs to ensure safe and efficient EACS procedures.

Our study’s emphasis on the learning curve of EACS aligns well with the growing interest in surgical simulation models to improve surgical training. An interesting development of recent years is the creation of several neurosurgical training simulators aiming to shorten the EACS learning period. Coelho et al^14^ developed a scaphocephaly and trigonocephaly treatment simulator, covering all aspects of the procedure, from patient positioning to performing osteotomies. A more recent study by Cuello et al^18^ described similarly a low-cost simulation model for endoscopic-assisted sagittal craniosynostosis repair. The results of their study showed that all participants improved their performance on the Global Rating Scale and reduced their number of errors and time required to perform the task after completing the training program.

The strengths of this study include the large sample size and a long follow-up period, which allowed for a thorough examination of the EACS learning curve. In addition, the study was performed at a single institution, which minimized the effects of varying surgical techniques and variability in patient care. All patients at the Radboudumc Center of Expertise for Craniofacial Anomalies underwent surgery using a multidisciplinary approach involving either one of the 2 craniofacial neurosurgeons or one of the 2 craniofacial maxillofacial surgeons.

However, there are some limitations to this study, Firstly, being a single-center retrospective study, the findings might not be generalizable across other centers. Secondly, although the study found a statistically significant learning curve for EACS surgery, it is important to note that statistical significance does not necessarily equate to clinical relevance. Further research is needed to determine the optimal number of surgeries needed to achieve the best outcomes.

## CONCLUSION

In conclusion, EACS is a safe procedure with a low complication rate of 7.4% over a 20-year period. Our study demonstrated a statistically significant learning curve associated with EACS. Each additionally performed EACS resulted in a 0.7% decrease in the odds of postoperative complications and a 0.8% decrease in the odds of requiring a blood transfusion. In addition, there was a significant reduction in the duration of hospital stay, duration of anesthesia and surgery time with increased surgical experience in EACS.

## Supplementary Material

SUPPLEMENTARY MATERIAL
