# Supplementary material for: Evaluating the Learning Curve and Patient Outcomes in Endoscopically Assisted Craniosynostosis Surgery: A 20-Year Analysis
Source: J Craniofac Surg. 2024 Oct 11;36(1):123–7. doi: 10.1097/SCS.0000000000010755 (PMC11658016; doi:10.1097/SCS.0000000000010755)
Supplement: SUPPLEMENTARY MATERIAL [file scs-36-123-s001.docx]

Supplemental tables

*Supplemental Table 1. Study population*

| **Variable** | **Number of patients (n)** | **Percentage of total study population (%)** |
| --- | --- | --- |
| **Gender**  *Male*  *Female* | 222  88 | 71,6  28,4 |
| **Cranioform**  *Multisuture*  *Scaphocephaly Brachycephaly*  *Trigonocephaly*  *Plagiocephaly* | 13  160  7  105  32 | 4,2  51,6  2,3  33,9  10,2 |
| **ASA** *I*  *II*  *III*  *IV* | 235  65  9  1 | 75,8  21  2,9  0,3 |
| **Syndrome present**  *Yes*  *No* | 18  292 | 5,8  94,2 |

*Supplemental Table 2. Linear regression analysis: influence of surgical experience on outcome measures with a continuous scale.*

| Dependent variable | Predictor variable | Coefficient  (B) | Standard Error (SE) | Standardized coefficient (Beta) | t-value | p-value | 95% confidence interval (CI) for B |
| --- | --- | --- | --- | --- | --- | --- | --- |
| Length of hospital stay | Surgical experience | -0,004 | 0,0005 | -0,455 | -8,962 | 0,0001 | (-0,005, -0,003) |
| Total blood loss | Surgical experience | -0,002 | 0,020 | -0,007 | -0,116 | 0,908 | (-0,041-0037) |
| Duration of anesthesia | Surgical experience | -0,053 | 0,016 | -0,188 | -3,362 | 0,001 | (-0,084, -0,0222) |
| Surgery duration | Surgical experience | -0,044 | 0,012 | -0,199 | -3,566 | 0,0001 | (-0,068, -0,020) |

Note: B= unstandardized regression coefficient, SE= standard error of the coefficient; Beta= standardized regression coefficient; t-value = t-test statistic; p-value = probability value; CI = confidence interval

*Supplemental Table 3. Binary regression analysis: influence of surgical experience on outcome measures with a dichotomous outcome.*

| Dependent variable | Predictor variable | Coefficient  (B) | Standard Error (SE) | Wald | df | p-value | Exp (B) | 95% confidence interval (CI) for Exp B |
| --- | --- | --- | --- | --- | --- | --- | --- | --- |
| Need of blood transfusion | Surgical experience | -0,008 | 0,002 | 11,649 | 1 | 0,001 | 0,992 | 0,988-0,977 |
| Post-operative complications | Surgical experience | -0,008 | 0,003 | 7,912 | 1 | 0,005 | 0,993 | 0,987-0,998 |
